# A widespread dissemination of *Bacillus licheniformis* in a tertiary hospital: an outbreak or pseudo-outbreak?

**DOI:** 10.1017/ice.2025.10223

**Published:** 2025-09

**Authors:** Secil Deniz, Ahmet Caliskan, Elif Seren Tanriverdi, Burhan Ozkan, Zeynep Ceren Karahan, Ilknur Kacar, Ayse Kivrak, Sirin Menekse

**Affiliations:** 1 Department of Infectious Diseases, School of Medicine, University of Pamukkale, Denizli, Turkey; 2 Department of Medical Microbiology, School of Medicine, University of Pamukkale, Denizli, Turkey; 3 Department of Medical Microbiology, Inonu University, Faculty of Medicine, Malatya, Turkey; 4 Department of Medical Microbiology, Ankara University, Faculty of Medicine, Ankara, Turkey; 5 Infection Control Unit, School of Medicine, University of Pamukkale, Denizli, Turkey; 6 Infection Control Unit, Koşuyolu High Specialization Education and Research Hospital, İstanbul, Turkey

## Abstract

**Objective:**

*Bacillus licheniformis* is a Gram-positive bacterium commonly found in soil and water. There have been very few reports on the pathogenicity of *B. licheniformis* in humans. In this prospective study, the emergence of cases affected by *B. licheniformis* during a period of 38 days was reported together with investigations into the sources of spread to hospitalized patients in a tertiary hospital.

**Methods:**

Blood cultures of 45 patients grew *Bacillus* spp. in October and November, 2021. To identify the source and prevent further dissemination of the pathogen, all commonly used materials were examined. Samples obtained from alcohol/water solutions yielded positive results for *Bacillus* spp., which pointed to the main distilled water tank of the hospital, subsequently found to be the main source. All isolates were sent for molecular analysis by arbitrarily-primed polymerase chain reaction (AP-PCR).

**Results:**

Molecular analysis with AP-PCR of 29 positive cultures showed a closely related clone of *B. licheniformis* in 25 specimens, including 23 blood samples and two distilled water samples. Considering the rarity of true infections with *B. licheniformis* and the mild clinical picture of the affected patients, the dissemination was considered to be a pseudo-outbreak.

**Conclusions:**

Prompt detection and elimination of any pathogenic spread and differentiation of a pseudo-outbreak from a true outbreak are of utmost importance in preventing unnecessary antibiotic prescriptions, diagnostic procedures, and interventions.

## Introduction

*Bacillus* species are Gram-positive, aerobic or facultative anaerobic endospore-forming bacilli commonly found in soil, water, dust, and plants.^1,2^ *Bacillus* spp. are generally considered contaminants rather than pathogens in the clinical environment. The exceptions to this include members of the *B. cereus* group (eg, *B*. *anthracis and B. cereus*).^1,3^ As spores have a high resistance to heat and many hospital disinfectants, including 70%–90% alcohol solutions, prevention of contamination with *Bacillus* spp. may be difficult in hospital settings.^4^

Among *Bacillus* spp., *B. licheniformis* has received particular biotechnological interest owing to its beneficial properties, distinct genetic makeup, and safety characteristics, offering a wide range of current and potential applications in the synthesis of bioactive compounds used in diverse industries such as agriculture, food production, biomedicine, and pharmaceuticals. For example, bacitracin, an antibacterial compound extensively used in the animal feed and veterinary medicine industries, is primarily synthesized from *B. licheniformis* and *B. subtilis*.^5–9^ Due to its non-toxigenic nature, *B. licheniformis* has been approved as a food additive worldwide, particularly as a probiotic.^10^ Moreover, a wide range of beneficial properties have been shown in animal studies. In two porcine models, it showed antiviral activity against porcine epidemic diarrhea virus,^11^ and improved barrier function in porcine intestinal epithelial cells, alleviating inflammatory responses against enterotoxigenic *Escherichia coli* F4 infection.^12^ In a rat model, it was shown to prevent and reduce anxiety-like and depressive-like behaviors.^13^

There have been very few reports on the pathogenic potential of *B. licheniformis* in humans, particularly in immunocompromised patients. Almost all these reports have been on either single cases caused by local predisposing factors such as injuries, accidents, use of catheters or pacemakers^1,14–19^ or small case series of two or more patients.^20,21^ To date, there have been no reports of *B. licheniformis* bacteremia confirmed by molecular analysis, emerging in the same period of time, and causing suspicions about a possible *B. licheniformis* outbreak or a pseudo-outbreak. Evidence-based differentiation between a pseudo-outbreak and an outbreak as early as possible would provide substantial benefits both on the part of patients and healthcare services, avoiding unnecessary treatments and excessive antibiotic use. In this prospective study, the emergence of apparent *B. licheniformis* cases during a 38-day period was reported together with investigations into the sources of spread to hospitalized patients.

## Materials and methods

### Participants

This prospective study was performed at Pamukkale University, School of Medicine in Denizli, Turkey. Over a 38-day period in October and November, 2021, *Bacillus spp.* grew from blood cultures collected from 45 patients admitted to 6 intensive care units and 13 clinical wards. During that interval, a total of 6,856 admissions and 25,709 hospitalization days, including pediatric patients, were recorded. Patients were enrolled based on the detection of one or more blood cultures positive for *Bacillus spp.*

The study was approved by the institutional review board, and all study protocols conformed to the Declaration of Helsinki. Preparation of the text complied with the infection control guidelines of the Outbreak Reports and Intervention Studies of Nosocomial Infection (ORION) Checklist.

### Hospital settings

Pamukkale University Hospital is a 900-bed tertiary referral center with 6 intensive care units (112 beds). The infection control team included two infectious diseases specialists, five dedicated nurses, and one microbiologist.

### Treatment of blood specimens

Blood samples were drawn after cleansing the skin with alcohol solution and were placed in blood culture bottles with antibiotic-neutralizing resin (Becton Dickinson Diagnostics, USA) whose caps were previously disinfected with alcohol. After collection, the samples were placed in the Bactec™ FX automated system (Becton Dickinson Diagnostics, USA). As part of routine laboratory workup, the samples flagged by the system were removed from the device, Gram stained, inoculated onto 5% sheep blood agar and MacConkey agar, and incubated for 24–48 hours at 35°C.

### Environmental investigations into possible sources

Environmental samples were collected from all possible materials related to patients’ care using sterile cotton swabs or injectors, which included 70% ethyl alcohol, plastic bottle caps, unused blood culture bottles (Becton Dickinson Diagnostics), commercial povidone-iodine solutions, gloves, saline solutions, unused injectors, and non-sterile cotton wools used during skin care. All liquid samples were directly sent to the laboratory and processed for culture in blood culture bottles. The swabs were inoculated onto trypticase soy broth and incubated for 48 h at 35°C. Following incubation, the samples were inoculated onto 5% sheep blood agar and MacConkey agar and incubated for 24–48 hours at 35°C. The liquid samples were processed in the same way as the blood cultures.

Blood and environmental samples with bacterial growth were then identified by the automated VITEK 2 Compact system (bioMérieux Vitek Hazelwood, MO). All bacterial samples were stored at –18°C.

At the time of detection of *Bacillus* spp., further specific typing was unavailable at our center; therefore, all the positive specimens were sent to another center where the matrix-assisted laser desorption/ionization time-of-flight mass spectrometry (MALDI-TOF MS) (bioMérieux, France) was available.

### Molecular analysis

Before submission for molecular analysis, all isolates confirmed by the MALDI-TOF MS were sub-cultured on 5% blood agar and sent to the Molecular Microbiology Laboratory of the Department of Microbiology at Inonu University, Malatya, to be analyzed by arbitrarily-primed polymerase chain reaction (AP-PCR).

After storage at –80°C, the DNA samples were genotyped on AP-PCR.^22^ Band profiles were analyzed using the GelCompar II software system (version 6.6; Applied Maths, Sint-Martens-Latem, Belgium). The similarity of band analysis was assessed using the Dice correlation coefficient. The UPGMA method (Unweighted Pairwise Grouping Mathematical Averaging) was used for cluster analysis.

**Figure 1.**
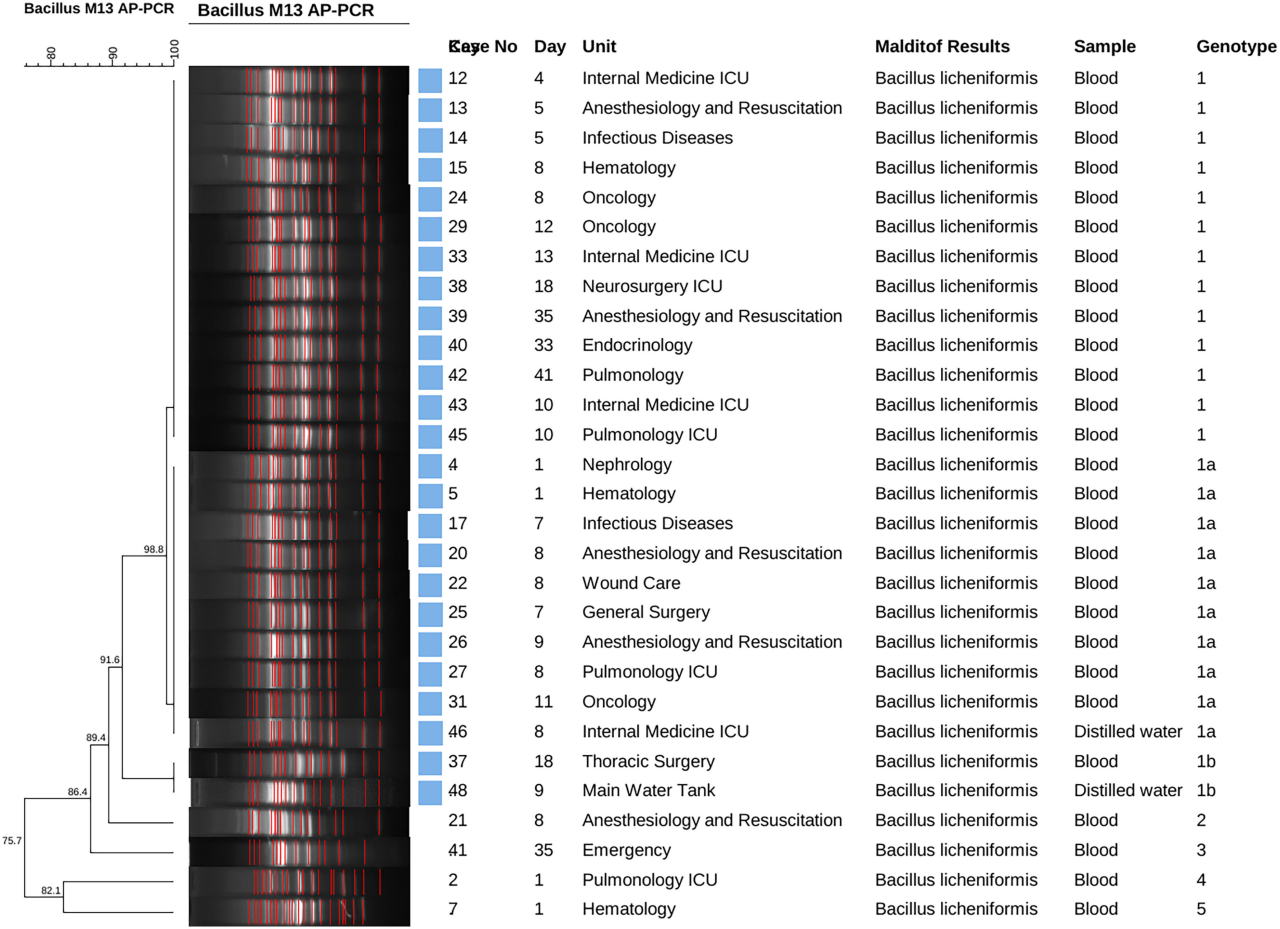
Arbitrarily-primed polymerase chain reaction results of *Bacillus licheniformis* isolates.

The genotypic classification of isolates was based on their genetic similarity as determined by the AP-PCR dendrogram results. Isolates producing bands of the same number and size were considered closely related and thus to be epidemiologically linked to the outbreak (Genotype 1–1). Those with minor differences were considered likely associated and were named Genotype 1–1a. Those with moderate differences were considered possibly linked and named Genotype 1–1b (Figure 1).

## Results

During the study period, a total of 6,162 blood culture bottles were collected, of which 79 were *Bacillus* (1.3%) and 913 were non-*Bacillus* (14.8%) growths. Blood cultures collected on four consecutive days from seven patients admitted to the internal medicine (n = 1) and pulmonary (n = 1) ICUs, hematology (n = 3), nephrology (n = 1) wards and emergency department (n = 1) yielded positive results for *Bacillus* spp. by the VITEC system. Of these positive cultures, three were obtained from patients on hospital Day 1. The diversity of the hospital settings and the detection of a common organism on the first hospital day aroused suspicion for a hospital outbreak or pseudo-outbreak from a common source. Upon detection of an increased number of *Bacillus* spp. within only four consecutive days as compared with sporadic detection in the past, the infection control team was alerted about a possible outbreak by the electronic notification system. On Day 5, four additional patients were detected to have growth of *Bacillus* spp. in blood cultures.

To identify the source and prevent further dissemination of the pathogen, a list of commonly used materials was prepared, including intravenous fluids, medications, and laboratory and culture equipment. All samples were sent for culture. On Day 8, blood samples of four additional patients were found positive, totaling 15 patients. The same day, *Bacillus* spp. was recovered from one sample of an aqueous alcohol solution used in the internal medicine ICU.

At our institution, commercial 96% ethyl alcohol is diluted with distilled water to obtain 70% ethyl-alcohol solution with activity against bacteria and fungi. It is stored in multi-use vials to be used for disinfection of the skin and the caps of blood-culture bottles. This procedure was individually carried out in each department at the discretion of nurses. The remaining environmental samples were negative. Further scrutiny was directed toward alcohol/water solutions. A brief investigation by the infection control team revealed that the distilled water used for the preparation of 70% ethyl alcohol solutions was obtained from the hospital water supply system. On Day 9, a sample was obtained directly from the main distilled water tank, which yielded a positive result for *Bacillus* spp., strongly suggestive of a pseudo-outbreak. The main distilled water tank is a component of a combined system used to produce distilled water from city water. The distilled water is stored in the main tank and distributed through water pipes throughout the hospital for various purposes, including the preparation of distilled water/alcohol solutions and hemodialysis.

All hospital services were alerted and warned to discontinue the use of the hospital distilled water. Further investigation revealed that the problem arose from shortcomings in the technical maintenance of the water tank, including filter replacement and chlorination. To ensure a safe water supply, all technical issues concerning the water tank were reviewed, filters were replaced and the automatic chlorination system was fixed. As an additional measure, all unused prepared alcohol-water solutions were removed. Dilution of ethyl alcohol was henceforth performed using only commercial sterilized distilled water. Despite all measures to eliminate the source of contamination, 15 new patients with *Bacillus* spp. bacteremia were detected up to Day 14 and 11 more up to Day 22. The source of new cases could not be identified. After an infection-free period of about 10 days, four more patients were documented from four diverse wards. Eventually, a comprehensive review of the environmental sources utilized for the preparation of the ethyl alcohol-water solutions revealed a plastic non-sterile mug used to mix alcohol with commercial sterile distilled water. All non-sterile plastic materials used for this purpose were removed from all hospital settings without testing by culture. Thereafter, alcohol-water solutions were prepared only in the pharmacy department of the hospital in aseptic settings and distributed to other departments. After the detection of the last four cases, no new cases were detected and the pseudo-outbreak was considered to be completely controlled. Overall, *Bacillus* spp. was detected in blood cultures of 45 patients admitted to 19 diverse hospital settings, including six intensive care units and 13 clinical wards (Table 1).

**Table 1.** Diverse hospital settings where *Bacillus* spp. and *B. licheniformis* were isolated

	*Bacillus* spp. (n = 45)	*B. licheniformis* (n = 27)
Anesthesia and Resuscitation ICU	12	5
Hematology	5	3
Internal Medicine ICU	4	3
Oncology	4	3
Pulmonology ICU	3	3
Infectious diseases	2	2
General surgery	2	1
Emergency	2	1
Neurosurgery ICU	1	1
Endocrinology	1	1
Gastroenterology	1	0
Thoracic Surgery	1	1
Pulmonology	1	1
Cardiac Surgery ICU	1	0
Cardiac surgery	1	0
Wound care	1	1
Neurology ICU	1	0
Urology	1	0
Nephrology	1	1

ICU: Intensive care unit.

During the isolation period, all clinical departments were visited, interviews were made with all staff concerned, instructions concerning the elimination of contamination were given and training programs were scheduled.

### Treatment

Among the first 11 patients whose blood cultures were positive for *Bacillus* spp., five were admitted the same day and two did not have fever. The remaining four patients developed fever during hospitalization while undergoing treatment for diverse conditions. These four patients received empiric vancomycin until the distilled water system of the hospital was found to be the culprit. After detection of the hospital distilled water system as the source of contamination and considering the prolonged median time to culture growth (20.2 hours), patients who had positive cultures for *Bacillus* spp. were considered to be part of a pseudo-outbreak and received no *Bacillus*-specific therapy.

### Strain identification and molecular analysis

**Figure 2.**
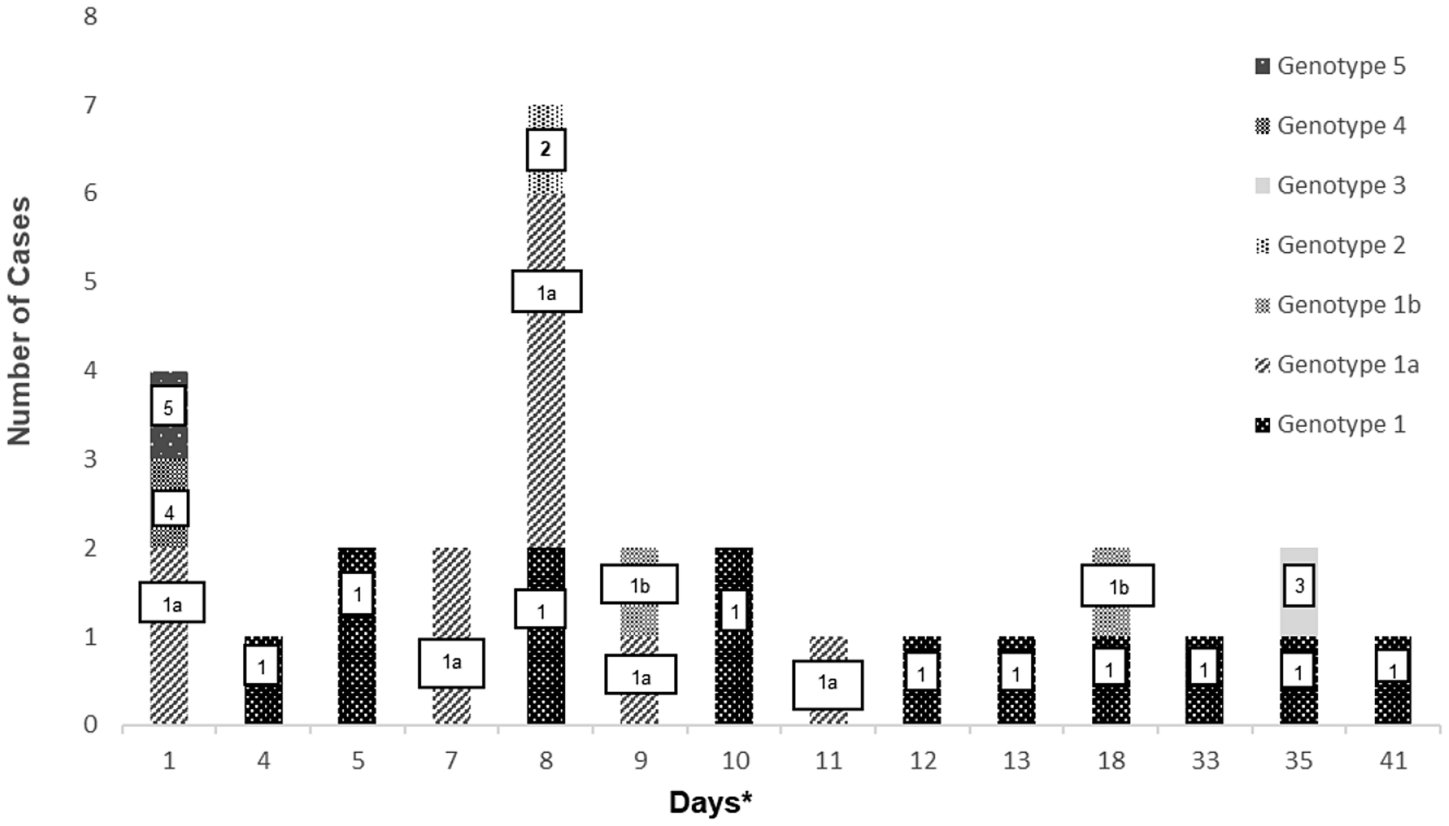
Epidemiological curve with clonal characteristics. *Days indicate the time at which blood cultures were obtained from patients.

All the positive specimens (n = 47), including two distilled water samples from the hospital systems, were sent to a dedicated center for identification with MALDI-TOF MS, where *B. licheniformis* was identified in 27 blood cultures and two distilled water samples from the hospital systems (Figure 2 The remaninig 18 positive blood cultures were read as *B*. *pumilus* (n = 5), *B*. *siralis* (n = 3), *B*. *clausii* (n = 2), *B*. *circulans* (n = 1), *Paenibacillus lactis* (n = 3), *Bacilllus* spp. (n = 2), *Enterococcus faecium* (n = 1), and an unidentified species (n = 1). Species other than *B. licheniformis* were not included in this analysis because of the species and genotypic match between blood and water samples. Molecular analysis with AP-PCR of 29 *B. licheniformis* isolates showed a closely related strain of *B*. *licheniformis* in 25 specimens, including 23 blood samples and the two distilled water samples. The other four blood specimens belonged to four distinct *B. licheniformis* strains.

### Patient characteristics

Overall, *B. licheniformis* was isolated in blood samples of 27 patients. Sociodemographic, clinical, and laboratory characteristics of the patients are summarized in Table 2. The median age of the patients was 59 (IQR 39–68) years. The median time to *B. licheniformis* isolation from hospital admission was 5 (IQR 0–11) days. Seven patients (25.9%), all of whom had no history of previous admission, tested positive for *B. licheniformis* on their first day of hospitalization.

**Table 2.** Sociodemographic, clinical, and laboratory characteristics

Case no.	Age/ Sex	Complaints & morbidities	Fever	WBC (Cells/µ L)	CRP (mg/L)	PCT (ng/mL)	Isolation day	Genotype
**2**	23/F	COVID-19	–	6.570	17.7	0.1	0	**4**
**4**	50/F	Renal transplantation & peritonitis	+	10.980	60.0	0.6	0	**1a**
**5**	54/M	Hematologic malignancy	+	1.100	55.8	0.1	10	**1a**
**7**	68/M	Hematologic malignancy	–	1.430	6.4	0.6	0	**5**
**12**	68/F	Solid-organ malignancy	–	25.080	133.5	0.6	1	**1**
**13**	62/M	Solid-organ malignancy	+	0.30	297.6		5	**1**
**14**	58/F	DM, HT, Pyelonephritis	–	22.000	65.5	0.2	3	**1**
**15**	59/F	HT, Solid organ & hematologic malignancy	–	0.71	62.6	0.1	19	**1**
**17**	25/M	Fabry disease	–	13.730	214.0	0.1	0	**1a**
**20**	81/M	DM, HT, BPH	–	4.280	219.7	4.1	10	**1a**
**21**	25/F	Multi-trauma**	+	6.620	118.0	24.5	24	**2**
**22**	73/M	Diabetic foot infection	–	11.980	11.6		0	**1a**
**24**	57/M	Solid organ malignancy	–	9.810	250.7	0.7	11	**1**
**25**	38/F	Solid organ malignancy	–	9.580	282.4	0.1	4	**1a**
**26**	63/M	Solid organ malignancy	–	10.490	103.8	8.1	0	**1a**
**27**	70/M	Solid organ malignancy	–	14.880	53.3	4.3	16	**1a**
**29**	78/M	Solid organ malignancy	–	6.700	98.7	0.9	1	**1**
**31**	39/F	Synovial sarcoma	+	9.940	181.5	0.3	7	**1a**
**33**	75/M	Hematologic malignancy	+	5.850	202.0	0.3	50	**1**
**37**	26/M	Sarcoma	–	12.860	169.0		16	**1b**
**38**	85/F	Solid-organ malignancy	+	12.420	41.7	1.4	25	**1**
**39**	60/M	COVID-19*	+	20.460	19.9	2.0	7	**1**
**40**	43/M	Diabetic foot infection	–	9.800	12.7	0.1	4	**1**
**41**	2/M	Meningitis	+	16.710	202.0	1.8	0	**3**
**42**	51/M	Solid organ malignancy	–	7.550	19.0	0.1	4	**1**
**43**	64/M	Solid organ malignancy	–	11.310	201.0		2	**1**
**45**	61/M	Renal transplantation & COVID-19	+	9.020	132.0	0.6	1	**1**

Case no corresponds to the case numbers shown in Figure 1.

DM: Diabetes mellitus, HT: Hypertension, BPH: Benign prostatic hyperplasia, WBC: White blood cell, CRP: C-reactive protein, PCT: Procalcitonin.

Reference ranges: WBC: (4–10 Cell/µ L), CRP: (<5 mg/L), PCT: (<0.5 ng/mL).

*: Concomitant isolation of *Klebsiella pneumoniae*; **: Concomitant isolation of *Candida parapsilosis.*

## Discussion

To our knowledge, this is the first documentation of *B. licheniformis* detected in a cluster of patients. This pseudo-outbreak occurred over 38 days, involving 27 patients, the vast majority of whom showed no signs of infection. Initially, the emergence of *Bacillus* spp. from a wide range of hospital settings aroused suspicion for a hospital outbreak. Prompt investigations into the source demonstrated the hospital distilled water system to be contaminated. Considering that the first seven *Bacillus* spp.positive patients were detected in five different clinics, and that three positive cultures were obtained on hospital Day 1, our interest was also directed to the possibility of a pseudo-outbreak. Identification of *B. licheniformis* by the MALDI-TOF MS in both blood and environmental water samples supported our assumption in favor of a pseudo-outbreak. Further molecular analysis confirmed the bacterium to be of hospital distilled water system origin.

Our experience with this pseudo-outbreak of *B. licheniformis* emphasizes the need to review possible sources of contamination, which might otherwise have been overlooked in source-limited developing countries. In our case, it was the way in which alcohol solutions were individually prepared by each service or department using the distilled water from an inadequately maintained hospital water system. This accounts for the detection of *B. licheniformis* from a wide diversity of clinical settings (Table 1). Unfortunately, apart from that about hand hygiene antiseptics, there has been no guidance document in our country about how to prepare these solutions aseptically. To our knowledge, this is the first report of a pseudo-outbreak caused by *B. licheniformis* proliferated in alcohol preparations widely used in hospital settings of developing and source-limited countries where commercial ready-to-use antiseptics are not always available. In clinical practice, alcohol is primarily used for antiseptic purposes, but the ineffectiveness of alcohol solutions against *Bacillus* spp. is highly underestimated.^23^ Some authors recommend using 2% chlorhexidine gluconate added in 70% isopropyl alcohol to increase the effectiveness of skin antiseptics.^24^

The presence of fever may be a confounding finding and may make it difficult to differentiate between an infection and contamination, particularly in patients with malignancies, which may or may not lead to fever. In this patient series, the presence of fever in four patients was initially considered in favor of an infection and was treated with empirical antibiotic therapy until the distilled water system of the hospital was found to be the culprit. This emphasizes the need for early identification of a pseudo-outbreak to avoid excessive use of antibiotics. In our study, the time from the first identification of positive blood cultures to the determination of the source *of Bacillus* spp. was five days. Another point of consideration is that, due to unavailability of MALDI-TOF MS, the typing of species could only be made at a foreign center about one month later after the elimination of positive cultures, at which time we could realize that a considerable proportion of blood samples (18 patients, 40%) showed strains other than *B*. *licheniformis*. The wide diversity of these *s*pecies during a period of 38 days and the absence of positive environmental samples drived us away from the possibility of another pseudo-outbreak. Moreover, we traced these patients back and found that positive blood cultures had been obtained on the first two days of admission in 11 patients, suggesting community-acquired transmissions.

## Conclusion

The detection of rare bacterial organisms known to be infectious in any hospital setting should arouse suspicion for an outbreak and alarm the hospital surveillance and control team. Differentiation of a pseudo-outbreak from a true outbreak is of utmost importance in preventing unnecessary antibiotic prescriptions, diagnostic procedures, and unnecessary interventions.
